# Anticancer Potential of the Cyclolinopeptides

**DOI:** 10.3390/cancers15153874

**Published:** 2023-07-30

**Authors:** Adnan Fojnica, Zehra Gromilic, Semir Vranic, Michael Murkovic

**Affiliations:** 1 Molecular Biology and Biochemistry, Gottfried Schatz Research Center, Medical University of Graz, 8036 Graz, Austria; fojnica@student.tugraz.at; 2 Institute of Biochemistry, Graz University of Technology, 8010 Graz, Austria; zehra.gromilic@student.tugraz.at; 3 College of Medicine, QU Health, Qatar University, Doha 2713, Qatar; svranic@qu.edu.qa

**Keywords:** flaxseed, cyclolinopeptides, cancer, potential therapeutics

## Abstract

**Simple Summary:**

Peptides are ubiquitous molecules abundantly found in nature, and their diverse functions and biological activities have been extensively examined over the years. In the past, in vitro and in vivo biological effects of flaxseed oil have been well-examined and reported in numerous studies. However, the effects of many individual components from the flaxseed are yet to be examined. Cyclolinopeptides (CLPs), one of the components, have gained significant attention due to their cyclic nature and hydrophobic properties. CLPs are isolated from flaxseed and typically consist of eight, nine, or ten amino acid residues. Experimental investigations into the biological activities of CLPs began in the 1980s and early 1990s, revealing their potential therapeutic applications. In recent years, particularly, there has been interest in examining the anticancer effect of CLPs. Diverse bioactivities and potential therapeutic CLPs aspects seek novel approaches and further investigation to maximize utility.

**Abstract:**

Novel therapeutic agents to combat cancer is an active area of research, as current treatment options have limitations in efficacy and tolerability. One of these therapeutic agents in our immediate environment is cyclolinopeptides (CLPs). CLPs have several advantages that make them suitable for daily consumption and potential therapeutics in cancer research. They are natural compounds, having high specificity, low toxicity, low cost, and an overall simple extraction process. Over the years, numerous in vitro studies in cancer cells demonstrated CLPs to possess anti-proliferative, apoptotic, and anti-angiogenic effects, as well as the ability to induce cell cycle arrest and inhibit cancer cell growth in various cancer types, including breast cancer, gastric cancer, and melanoma. This paper provides an overview of the significance and potential of CLPs as therapeutic agents, emphasizing their promising role in cancer treatment based on different cancer cell lines. The mechanism of action of CLPs in cancer cells is multifaceted. It involves the modulation of multiple signaling pathways, including inhibition of protein kinases, modulation of apoptosis-related proteins, and regulation of oxidative stress and inflammation.

## 1. Flaxseed and Its Constituents

**Figure 1:**
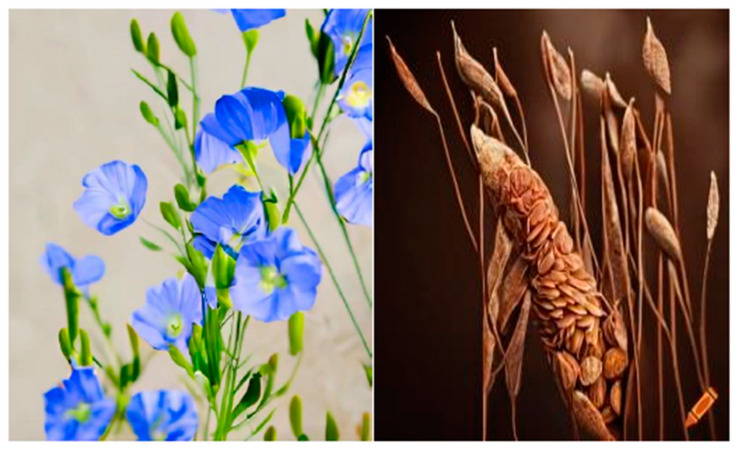
Flowering *Linum usitassimum* on the left and dried seed of the flax on the right. Images generated by AI software: Craiyon.

Flaxseed, also known as linseed (*Linum usitatissimum* L.), is a spring flowering annual plant flax cultivar belonging to the Linaceae family [1]. Flax usually grows up to 100 cm and has a long main stem and a short taproot, while root branches extend to 120 cm in soil. In thin stands, branches develop from the plant base, while in thick seeding, only the main stem develops. The flax fruit is a five-celled capsule, usually containing ten seeds [2]. Throughout history, selective breeding led to highly branched, shorter varieties with improved seed yields. Approximately 200 *Linum* species have been identified worldwide [2,3]. The plant is mainly cultivated to produce fibers for making linen, while plants grown for oilseed are of the same species but belong to different cultivars [2]. Flax varieties grown for seed production are highly branched, shorter, and produce a high yield of seeds, whereas plants cultivated for fiber utilization mature early and have tall stems with little branches (Figure 1). Common fiber flax is harvested before the seed is mature, as the fiber quality is at its best in the premature phase. On the other hand, seeds during this phase produce high-quality oil; part of the seeds are used for replanting, and the rest is sold in the oilseed market.

The terms “flaxseed” and “linseed” are often used interchangeably in the common language; however, there is a significant difference in the scientific literature. Generally speaking, linseed is used to describe flax used for feed purposes and industry, while flaxseed is used to describe flax used for human food consumption [4].

Flaxseed is cultivated in more than 50 countries all around the globe [1]. Canada is the leading producer of flaxseed oil, with 1,260,000 tons annually, following USA and China [1]. European countries in which flax is primarily cultivated are the Czech Republic, Slovakia, France, Germany, Italy, Belgium, and Ireland [1]. Mature flaxseeds are oblong and flattened in shape and measure approximately 2.5 × 5.0 × 1.5 mm [5]. The weight of one seed is approximately 5 mg, with a positive correlation between seed weight and oil content [5].

Typically, flaxseed contains 41% fat, 28% fiber, 20% protein, 7.7% moisture, and 3.4% ash, indicating that the percentage varies depending on the cultivar, growing conditions, and seed processing [6]. Other components commonly found in flaxseed are cyclolinopeptides (CLPs), lignans, phenols, phytic acid, cyanogenic glycosides, trypsin inhibitors, linatine, condensed tannins, cadmium, selenium, and vitamins and minerals [7,8].

## 2. Cyclolinopeptides

Cyclolinopeptides (CLPs) represent a group of cyclic, hydrophobic peptides usually composed of eight (octapeptides), nine (a nonapeptide), or ten (decapeptide) amino acid residues, having molecular weights in the range of 950–2300 Da [9,10]. Flaxseed orbitides, or linusorbs, may be used interchangeably to refer to CLPs. However, often they will have distinct and broader meanings, particularly in the case of orbitides [9,10].

The very first cyclic peptide ever identified was CLP-A. The peptide was extracted in 1959 by Kaufmann and Tobschirbel from the deposited sediments of crude flaxseed oil [9,10]. Subsequently, each newly discovered cyclic peptide from flaxseed was named by the next letter in the alphabet. Thirty-nine CLPs primarily containing proteinogenic and oxidized amino acids were identified in flaxseed oil, roots, and seeds [1,9,10].

The highest concentration of CLPs is found in cold-pressed flaxseed oil, and usually, up to around 15 CLPs are identified in the flaxseed oil using common analytical tools [1]. Flaxseeds are crushed and passed through cold press oil expellers without employing any pre-press heat treatment prior to the mechanical pressing stage to extract flaxseed oil and achieve elevated levels of CLPs. Extracted cold-pressed oil can be directly used as a food source after mechanical treatment.

Detailed representations of the peptides and their primary structures are shown in Table 1. Detailed chemical representation, as well as the nucleotide and protein sequence, were provided by Shim and her group in 2019 [10].

## 3. Biological Activity of CLPs

Peptides are among the most present molecules in nature, and over the years, a special focus has been put on their function and biological activities. The natural function of CLPs in plants is still unknown [1]. However, the biological activity and health benefits of flaxseed oil, fibers, and meals have been well-established over the last few years [4]. Mainly, the biological activity of flaxseed oil is related to the exceptionally high amount of α-linolenic acid (ω-3 fatty acid) and its important role in cardiovascular and other diseases [11,12]. However, more and more biological activities of hydrophobic CLPs have been identified, and immune-system-suppressing and cancer-inhibiting effects were reported [1,10,13,14].

Experiments showing the biological activities of CLPs started in the nineteen eighties and early nineties. In 1986, it was reported that CLP-A inhibits cholate uptake into hepatocytes [15]. A possible explanation of this effect is a tripeptide block of two phenylalanine (Phe) and proline (Pro). This structure is found in CLP-A, which, once present in other structures (e.g., somatostatin), suppresses the transport system of hepatocytes [15].

A few years later, CLP-A was tested for immunosuppressive activity using a plaque-forming cell (PCF) test. In this test, CLP-A affected both primary and secondary humoral immune responses (HIR) [16]. In the graft versus host reactions, delayed-type hypersensitivity (DTH), and allograft rejection experiments, CLP-A caused prolonged skin rejection time and a reduced reaction index [16].

In 2001, CLP-A’s immunosuppressive effect was compared to cyclosporine A—a well-studied and clinically used immunosuppressant [17]. The experiment showed a similar mechanism in both cyclic structures, inhibiting the action of interleukin-1 and interleukin-2. Additionally, the induction of T and B cell proliferation and immunoglobulin synthesis by CLP-A was observed in the same experiment [17]. Besides CLP-A, Morita and co-workers reported CLP-B as a suppressing mitogen-induced response of human peripheral blood lymphocytes [18]. CLP-B inhibited lymphocyte proliferation induced by mitogen in mice [18]. CLP-A, CLP-C, and CLP-E induced cell apoptosis in human lung epithelial cancer cells [19]. Similar findings were reported for CLP-K and CLP-J [18,20].

Bell and colleagues demonstrated the antimalarial activity of CLA through the inhibition of human malaria parasites (*Plasmodium falciparum*) in culture [14]. Even though a clear correlation between the structure of the cyclolinopeptide and the inhibition of human malaria parasites in cell culture could not be found, a strong correlation between the hydrophobic nature of CLP-A and the antimalarial activity was evident [14]. The inhibition of malaria parasites increased with more hydrophobic amino acids in the cyclic peptide chain [14].

Another interesting observation was reported by Shim and her group [21]. CLPs can induce the production of heat shock protein in *Caenorhabditis elegans*. The treatment of nematodes in culture with CLP-A stimulates the production of proteins up to 3.5-fold, with a recommended concentration of CLP-A of 1.0 µM [21].

In 2010, Rempel and co-workers observed CLP-A binding to human serum albumin (HSA) [22]. HSA is the most abundant protein in blood plasma, and it is believed that the interaction of these structures would have an important utilization in nanotechnology and the biomedical sector. Interaction occurs through the hydrophobicity of Sudlow site I in HSA and CLP-A [22].

Another potential function of CLPs was reported in 2016 [23]. The group tried to examine the inhibitory effect of CLPs on osteoclast differentiation [23]. Almost all tested CLPs inhibited osteoclast differentiation at specific concentrations without damaging cell viability; TRAP activity was used to indicate osteoclast differentiation. However, the best IC50 (drug concentration necessary to produce the desired effect) results were obtained for CLP-I, CLP-O, CLP-F, CLP-N, and CLP-H peptides. All CLPs with significant activity had tryptophan in their cyclic structure; accordingly, CLPs with lower activity were missing tryptophan [23].

Many biological activities have been reported over the years. However, in this review, we will specifically focus on the anticancer effects of CLPs and thoroughly examine the available literature.

## 4. Literature Search Methods and Search Strategies

During the initial phase, a comprehensive search was conducted on the PubMed/MEDLINE database to gain a general understanding of the topic. No specific filters were applied during this search. In the subsequent phase, the search was refined to include specific scientific terms, such as “cyclolinopeptides”, “CLPs”, “flaxseed orbitides”, “flaxseed peptides”, “flaxseed cyclic peptides” or “linusorbs”, in combination with keywords, such as “tumor”, “cancer”, “anticancer”, “antitumor”, “neoplasia”, or “treatment”.

## 5. Cancer Research and Cyclolinopeptides

Cancer is the second leading cause of death that continues to be a major global health challenge, accounting for nearly 10 million deaths annually [24]. The main hallmarks of cancer cells, such as uncontrolled growth and the ability to invade surrounding tissues, lead to the formation of malignant tumors and spreading through the body [25]. Despite marked progress in cancer research and treatment, cancer still poses a significant burden in terms of sickness and mortality on a global scale. Therefore, searching for novel therapeutic agents to combat cancer is an active area of research, as current treatment options have limitations in efficacy and tolerability. One of these therapeutic agents found in our immediate environment is CLPs [5,13,20].

CLPs have several advantages that make them suitable for daily consumption and potential therapeutics in cancer research. They are natural compounds, having high specificity, low toxicity, low cost, and an overall simple extraction process [20,21,26]. Over the years, numerous in vitro studies in cancer cells demonstrated CLPs to possess anti-proliferative, apoptotic, and anti-angiogenic effects, as well as the induction of cell cycle arrest, and inhibition of cancer cell growth in various cancer types, including breast cancer, gastric cancer, and melanoma [21]. The mechanism of action of CLPs in cancer cells is multifaceted. It involves the modulation of multiple signaling pathways, including inhibition of protein kinases, modulation of apoptosis-related proteins, and regulation of oxidative stress and inflammation [21].

Furthermore, CLPs have been shown to possess immunomodulatory properties, enhancing the body’s immune response against cancer cells. Additionally, CLPs have been reported to have similar effects to conventional chemotherapy drugs, suggesting their potential use as adjuvants in cancer treatment [21].

The value of investigating CLPs in the context of cancer is their potential as a novel and promising therapeutic option. CLPs offer several advantages, such as being highly specific natural compounds, making them attractive candidates for cancer therapy. CLPs were also subjected to toxicological evaluation, and no toxic effects were found in rats and mice when administered orally or intravenously [21]. Further studies are needed to evaluate the safety of these peptides in humans. Understanding the underlying mechanisms of CLPs’ anticancer effects can contribute to developing targeted therapies that are effective against cancer cells while sparing normal cells, minimizing adverse effects associated with conventional cancer treatments.

Additionally, complementary and alternative medicine (CAM) plays a crucial role in healthcare by offering patients a holistic approach to care, diverse therapeutic options, and the potential to address treatment gaps without side effects [27,28]. The idea of entirely natural, “plant-grown drugs“ has been recognized as attractive to many people around the globe [27,28]. In Europe, 25.9% of the examined individuals have reported using natural compounds preferentially to either complement therapies or use them as a completely alternative approach [27,28]. Among the examined 21 European countries, Germany had the most reported cases of CAM use [27,28]. A similar trend is also observed in the USA, and CAM approaches have increased over the years [28,29].

So far, in clinical databases, there are no registered clinical trials dealing with applying CLPs to cancer patients. The only clinical trial (NCT01846117) that is registered regarding flax and its components in clinicaltrials.gov is the study regarding secoisolariciresinol diglucoside (SDG) [30]. A Canadian research team studied whether lignan intake reduces oxidative stress and inflammation through dietary intervention [30]. In addition, no reported study examines the effect of CLPs in cancer induction and experimental animal models. All the experiments carried out on CLPs are in vitro studies, wholly based on cell cultures [13,21,31,32,33,34,35,36]. Some studies regarding CLPs and anti-inflammatory effects primarily include mouse models [37,38]. Similarly, the anticancer potential of flaxseed oil is well-studied in mouse models [24,25,26,37,38,39]. Following general trends, clinical applications of the CLPs may wait several years for the first trials to start [40].

## 6. In Vitro Studies

To the best of our knowledge and according to the PubMed/MEDLINE database, the first study regarding the anticancer potential of the CLPs was conducted in 2015 in breast and melanoma cancer cell lines [33]. Our investigation on Google Patents uncovered a US patent application filed by Reaney and his research group in 2013 [19]. The patent application revealed their findings regarding the connection between CLPs (A, B, and E) and their potential to induce apoptosis in cancer cell lines [19].

In the last five years, research has been actively conducted on different cancer types, including gastric cancer [31,41], breast cancer [32,33,36], glioblastoma [35], adenocarcinoma [42], lung cancer [21], melanoma, and bone cancer [13]. Accordingly, a list of the most important studies and direct anticancer potential of the CLPs are summarized in Table 2 but also demonstrated through the rest of the manuscript.

### 6.1. Breast Cancer and CLPs

Breast cancer is the most common malignancy in women, and each minute three women are diagnosed with breast cancer worldwide [43]. The most frequently mutated genes in breast cancer cells are *TP53* and *PIK3CA* [43]. Owiti et al. [33] examined the influence of CLP-A, CLP-B, CLP-C, and CLP-E on breast cancer cell lines SK-BR-3 and MCF-7 and human melanoma cell lines A375 [33]. The objective of utilizing CLPs in the study was to achieve a specific effect by countering the growth and proliferation of cells caused by mutations in certain genes by inducing cell cycle arrest and/or promoting the overexpression of tumor suppressor genes. Concentrations that were tested were 25 μg/mL, 50 μg/mL, 100 μg/mL, 200 μg/mL, and 400 μg/mL, and the effect was observed after the 24 h and 48 h of exposure [33]. In the MCF-7 breast cancer cell lines, the highest cytotoxicity was observed for CLP-B (19%) and CLP-A (18%). While in the case of SK-BR-3 cell lines, the highest cytotoxicity was seen in the cells treated with the CLP-A (75%), following CLP-B (41%), CLP-C (36%), and finally CLP-E (28%) [33]. It is often reported that the cytotoxicity effect of CLPs reduces over time [33]. However, in this case, CLP-B, CLP-C, and CLP-E maintained their effect after 48 h [33].

In a study from 2019 [32], Jina Yang and her group examined the effectiveness of CLPs mixture in fighting human breast cancer. For this purpose, MDA-MB-231 and SK-BR-3 cell lines were investigated using the WST-8 assay. Cell lines were treated with the CLP-A and CLP-B, using concentrations of 25 to 400 μM [32]. The concentration-dependent decrease was observed across MDA-MB-231 cell lines. CLP-A exhibits significantly higher activity compared with CLP-B [32]. No significant cytotoxic effects were observed in the human breast cancer SK-BR-3 cells using CLP-A and CLP-B [32].

### 6.2. Melanoma and CLPs

The next cancer type for which the toxicity of CLPs was examined was melanoma [33,34]. Melanoma originates from the melanocytes and represents the most lethal form of skin cancer [44]. The dysregulated activation of the AKT pathway is common in melanoma and occurs in more than 70% of cases [44]. One of the aims of the peptide interaction in melanoma cells would be to modulate the AKT signaling pathway [41]. In the study, in vitro assays are applied on melanoma cells in order to examine the cytotoxicity effects of CLPs. No effect was observed for the first 24 h, by any of the four CLPs (A, B, C, and E), even at the highest concentrations [33]. After 48 h, the highest cytotoxicity effect was observed for CLP-A, following CLP-C and CLP-E. No cytotoxicity effect was observed for the CLP-B [33].

Another study that involved melanoma and the effect of CLPs on melanogenesis was conducted in 2022 [34]. For this purpose, mouse-derived B16-F10 melanoma cells were used. Contrary to expectations, CLP-A and CLP-B only reduced the pigmentation in the cells without any cytotoxic effect on melanoma cells. The same effect was observed once CLP-A, CLP-B, CLP-D, CLP-E, CLP-F, and CLP-G were mixed [34]. A possible explanation for this effect may be a low concentration of the applied CLPs, as only concentrations of 25 to 100 μg/mL have been used [34]. Some experiments conducted in the past showed that cytotoxic effects would start at concentrations of at least 200 μg/mL. The exposure time was identical to the previous experiments [33,34].

### 6.3. Gastric Cancer and CLPs

In a study from 2018, Xian-Guo Zou and his group examined the anticancer potential of CLP-A and CLP-B for gastric cancer [31]. Gastric carcinoma is the most common malignancy affecting the stomach [45]. It is the fifth most common malignancy in the world and accounts for the third most deadly neoplasm [45]. As the inactivation of tumor suppressor genes is one of the hallmarks of gastric cancer [46], one of the targets for the peptides would be to stimulate the expression of the tumor suppressor genes. The experiment used the gastric cancer cell line SGC-7901, and both CLP-A and CLP-B demonstrated significant antitumor cytotoxic activity. However, CLP-A had a significantly higher effect [31]. Of note, the type of CLPs and the respective concentration, in combination with exposure time, are considered essential parameters for such experiments. It is often the case that the cytotoxic effect will start after 24 h or 48 h [31]. Some CLPs will degrade over time and oxidize into another structure (e.g., CLP-B oxidizes into CLP-C), which can significantly impair their activity and change the effect [1]. Additionally, relatively low CLPs concentrations (80–240 μM) were shown to be sufficient to reduce the cell viability of SGC-7901 cells. A year after, the same authors [41] found that CLP-A and CLP-B induce cell cycle arrest in the examined gastric cancer cells. G1 phase cell cycle arrest was associated with the downregulation of CDK2, CDK4, cyclin D3, and cyclin E, and upregulation of the p21WAF1/CIP1 and p27KIP1 was observed for the gastric cells treated with both CLPs [41].

### 6.4. Giant-Cell Tumor of the Bone and CLPs

In 2019, the antitumor effects of the CLPs mixture were examined for the giant-cell tumor of the bone (GCTB) cell lines [13]. Most giant-cell tumors are benign; however, tumor cells have a potential for locally aggressive behavior and the capacity to metastasize [47]. A potential target for the CLPs effect in GCTB will be the RANK expression, as this pathway is involved in the pathogenesis and CLP-F [48,49]. Cell number decreased for all tumor cell lines treated with the mixtures of CLPs [13]. The exact mechanism was examined using flow cytometry and cell cycle assays [13]. The results indicated that antitumor effects originate from the inhibition of cell proliferation as DNA synthesis was suppressed, and the number of cells found in the G0/G1 phase drastically increased in cell lines treated with cyclic peptides [13].

### 6.5. Glioblastoma and CLPs

An anticancer effect of CLP-A and its underlying mechanism in glioblastoma cells was studied by Sung and colleagues [35]. As the most common and aggressive brain tumor, additional efforts are needed to understand this malignancy [50]. The cell motility of glioblastoma increases the resilience of GBM and makes it harder for treatment [51]. Once glioblastoma cells are treated with CLP-A, induced apoptosis, suppression of glioblastoma cell motility, and cell cytotoxicity are observed [35]. This observation is vital as a driving mechanism of cancer, metastasis, and organ invasion could be prevented through CLP-A treatment [35]. The underlying mechanism behind such an effect may be a suppressed activation of proto-oncogenic factors Stat3 and Src [35]. Tumor cell lines from human patients and cancer animal models were used for an experiment. Furthermore, in vivo studies would be required to confirm the significance of the observed effects [35].

### 6.6. Lung Cancer and CLPs

Shim and her group conducted a study regarding the effect of CLPs on the modulation of regulatory genes that induce apoptosis in Calu-3 cells (human lung cancer cell lines) [21]. For all three CLPs, concentrations of 0.1–100 μM were applied, and expression of p53 upregulated modulator of apoptosis and anti-apoptotic gene *BCL2* (anti-apoptotic gene decreased as the concentration of CLPs increased) [21]. Additionally, microarray assays show overexpression of several genes involved in the apoptosis process, such as *CIDEB, TNF, FAS,* and *TP53BP2,* for all three CLPs [21].

### 6.7. Adenocarcinoma and CLPs

Adenocarcinoma is a cancer type that arises from the glandular epithelium [52,53]. As *TP53* mutations are common in adenocarcinomas, biological molecules activating p53 and caspases are required [53]. Xian-Guo Zou et al. [42] conducted a study regarding applying CLP-A, CLP-B, and CLPs mixture (containing 13 CLPs) on the adenocarcinoma cell line. At 120 and 200 μg/mL concentrations, peptides activated p53 and caspase, increased Bax/Bcl-2 ratio, and inactivated AKT [42]. Most of the observed morphological changes will not occur before 36 h. As AKT significantly contributes to cell survival and resistance to tumor therapy, CLPs inactivation of AKT may be a key drug in overcoming tumor resistance [54].

### 6.8. Cytotoxic Effects

Isomerization of the peptides may lead to higher cytotoxic effects, which was confirmed by Jadhav and his group in 2021 [36]. Namely, natural (L-pair) CLP-A showed lower cytotoxicity than the synthesized D-analog [36]. A most notable difference was obtained after 48 h at 100 μg/mL, where cytotoxicity of 100% was observed in the synthesized D-analog and only 40% for natural CLP-A [36]. Interestingly, after 72 h of exposure to the synthesized peptides, cytotoxicity levels were identical for 100 and 200 μg/mL [36]. D-Analogs of other CLPs might significantly increase their toxic effect as well.

**Figure 2:**
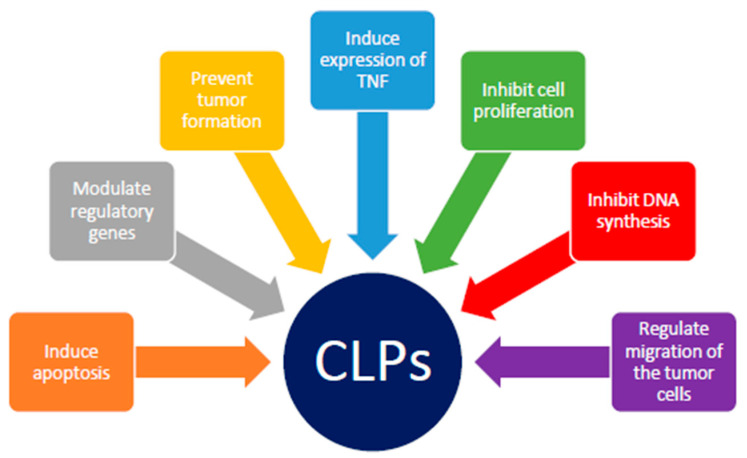
Diagram summarizing anticancer activities of various CLPs.

The available studies indicate that the effect of a relatively small proportion of peptides has been investigated over the years. Less than 20% of identified CLPs have been investigated. The diagram summarizing the activity of CLPs is shown in Figure 2, while the exact mechanisms and effects of each CLP are summarized in Table 3.

It is evident from the previous studies that CLP-A has received the most attention among the CLPs. This is mainly due to the high concentration of CLP-A in the flaxseed oil and low susceptibility to oxidation [1]. Active research has also been conducted on CLP-B and CLP-E. Other peptides are usually present in lower concentrations, which may be why their effects have not been extensively examined. Additionally, the effect of CLPs was observed only for a few cancer types [13,21,31,32,34,41]. For future experiments, we suggest that other common cancers are being tested (e.g., colorectal, prostate, and gynecologic cancers).

## 7. Conclusions and Future Perspectives

In the past, in vitro and in vivo biological effects of flaxseed oil have been well-examined and reported in numerous studies. However, the effects of many individual components from the flaxseed are yet to be examined. In the past ten years, primarily Canadian, South Korean, and Chinese research groups have been working on the effects of CLPs on different cancer cell lines. Research on the anticancer effects of CLPs holds a good therapeutic value for oncologic patients. The diverse bioactivities and potential mechanisms of action of CLPs make them an exciting area of investigation for developing novel cancer therapies.

Most of the experiments conducted over the years have focused on breast cancer cell lines. Even among these cell lines, research was limited to several common cancer cell lines; however, many other relevant cell lines that reflect the complexity and heterogeneity of breast cancer have not been tested. Interestingly, no studies examined the effect of CLPs on colorectal cancer, even though the colon and rectum play essential roles in the digestive system, and flaxseed/flaxseed oil may be consumed daily. Further research in this area, including well-designed preclinical, translational, and clinical studies, is required to fully elucidate the therapeutic potential of CLPs in cancer treatment.

The success of CLPs as a therapeutic agent would be dose- and cell-type-specific. In several studies, the lower dose of CLPs was observed to have a cytotoxic effect on cancer cells. However, an important thing to consider is the oral consumption of the peptides. Low CLP concentration may be efficient for cancer cell lines, so a significantly higher peptide concentration may be needed in oral consumption. Additionally, even though toxicity for the healthy tissue was not observed in most common rodent models (mice and rats), the application of higher concentrations might be potentially toxic for humans [21]. Therefore, additional efforts must be put in place for the wider application of the CLPs for human studies. Moreover, usage of the concentration of 100 µM was already shown to be lethal for lower eukaryotes.

Diverse bioactivities and potential therapeutic CLP aspects seek novel approaches and further investigation to maximize utility. Applications of the CLPs as a conjugated drug with other biomolecules should be considered for future studies.

## Figures and Tables

**Table 1:** Common CLPs found in the flaxseed oil extract are characterized by Hybrid Quadrupole Orbitrap Mass Spectrometry-RP HPLC (modified from our original article [1]).

Type	Sequence	Chemical Formula (MW-H^+^)
CLP-A	Ile-Leu-Val-Pro-Pro-Phe-Phe-Leu-Ile	C_57_H_85_N_9_O_9_ (1040.6543)
CLP-B	Met-Leu-Ile-Pro-Pro-Phe-Phe-Val-Ile	C_56_H_83_N_9_O_9_S (1058.6107)
CLP-C	Mso-Leu-Ile-Pro-Pro-Phe-Phe-Val-Ile	C_56_H_83_N_9_O_10_S (1074.6056)
CLP-D	Mso-Leu-Leu-Pro-Phe-Phe-Trp-Ile	C_57_H_77_N_9_O_8_S (1064.5638)
CLP-E	Mso-Leu-Val-Phe- Pro-Leu-Phe-Ile	C_51_H_77_N_8_O_9_S (977.5529)
CLP-F	Mso-Leu-Mso-Pro-Phe-Phe-Trp-Val	C_55_H_73_N_9_O_10_S_2_ (1084.4995)
CLP-G	Mso-Leu-Mso-Pro-Phe-Phe-Trp-Ile	C_56_H_75_N_9_O_10_S_2_ (1098.5151)
CLP-I	Met-Leu-Mso-Pro-Phe-Phe-Trp-Val	C_55_H_73_N_9_O_9_S_2_ (1068.5045)
CLP-K	Msn-Leu-Ile-Pro-Pro-Phe-Phe-Val-Ile	C_56_H_83_N_9_O_11_S (1090.6006)
CLP-L	Met-Leu-Val-Phe-Pro-Leu-Phe-Ile	C_51_H_76_N_8_O_8_S (961.5580)
CLP-M	Met-Leu-Leu-Pro-Phe-Phe-Trp-Ile	C_57_H_83_N_9_O_8_S (1048.5689)
CLP-N	Met-Leu-Met-Pro-Phe-Phe-Trp-Val	C_55_H_73_N_9_O_8_S_2_ (1052.5096)
CLP-O	Met-Leu-Met-Pro-Phe-Phe-Trp-Ile	C_56_H_75_N_9_O_8_S_2_ (1066.5253)
CLP-P	Met-Leu-Mso-Pro-Phe-Phe-Trp-Ile	C_56_H_75_N_9_O_9_S_2_ (1082.5202)
CLP-T	Mso-Leu-Met-Pro-Phe-Phe-Trp-Val	C_55_H_73_N_9_O_9_S_2_ (1068.5045)

Abbreviations: Ile—isoleucine, Leu—leucine, Val—valine, Pro—proline, Phe—phenylalanine, Trp—tryptophan, Met—methionine, Msn—methionine sulfone, and Mso—methionine sulfoxide.

**Table 2:** List of the most important studies regarding the anticancer activity of the peptides.

Type of Cancer	Cyclolinopeptides	Effect	Key Findings of the Study	References
Gastric cancer	CLP-A CLP-B	CLP-A and CLP-B induce apoptosis in gastric cancer cells	Effects of both CLP-A and CLP-B are highly dependent on the concentration of the peptides and the exposure time.	[31]
Lung cancer	CLP-A CLP-B CLP-E	CLP-A, CLP-B, and CLP-E induce apoptosis in lung cancer	Besides inducing apoptosis in human lung cancer, CLPs can induce HSP 70A production in *C. elegans*.	[21]
Bone tumor	CLPs mixture	CLPs mixture inhibits the synthesis of DNA in GCTB.	Cell viability decreases in a concentration-dependent manner.	[13]
Breast cancer	CLP-A CLP-B	CLP-A and CLP-B had a cytotoxic effect on the breast cancer cells	CLP-A and CLP-B had cytotoxic effects on triple-negative-subtype of breast cancer, even at low nanomolar concentrations.	[32]
Melanoma and breast cancer	CLP-A CLP-B CLP-C CLP-E	CLP-A, CLP-C, and CLP-E had cytotoxic effects on melanoma cells, while all four peptides had cytotoxic effects in the case of breast cancer cell lines.	The approximate effect for orally administrating CLP-A and other peptides is expected to occur after injecting 400–500 μg/mL of serum.	[33]
Melanoma	CLP-A CLP-B CLP-D CLP-E CLP-F CLP-G	CLP-A, CLP-B, and a mixture of six peptides did not cause cytotoxicity in melanoma cells. Peptides reduce mRNA levels of melanin production-related genes and the synthesis of melanin.	The 25 μM CLP-A and CLP-A concentrations lead to suppressed MITF and phosphorylated proteins such as CREB and PKA.	[34]
Glioblastoma	CLP-A	CLP-A suppresses Src gene activity and promotes cell apoptosis. CLP	CLP-A can suppress the motility of C6 cells as a critical factor for tissue invasion, cancer cell migration, and metastasis.	[35]
Adenocarcinoma	CLP-A CLP-B CLPs mixture	CLPs induce apoptosis	Individual CLPs and CLPs mixture release Cyt C, increase the level of Bax/Bcl-2 and stimulate the expression of caspase 3 and 9, eventually leading to apoptosis.	[42]
Breast cancer	L and D forms of CLP-A, CLP-D, and CLP-G	CLP-A, natural and synthesized variants inhibited the growth of the breast cancer cells	Isomerization of the peptides has a significant influence on its cytotoxic effects.	[36]
Gastric cancer	CLP-A CLP-B	CLP-A and CLP-B trigger G1 cell cycle arrest and prevent cell proliferation	CLP-A and CLP-B dose-dependently induce cell cycle arrest in gastric cancer	[41]

**Table 3:** Effects and mechanisms of CLPs in different cancer cell lines.

Effects	Cyclolinopeptides	Mechanisms	References
Induce apoptosis	CLP-A, CLP-B, CLP-C, CLP-E	CLP-A, CLP-B, CLP-C, and CLP-E may induce cell apoptosis by elevated expression of one of the following: HRK, FAS, CASP10, CIDEA, and CIDEB. Additionally, CLP-A downregulates the anti-apoptosis protein Bcl-2 and upregulates the pro-apoptotic protein Bax.	[21,31]
Modulate regulatory genes	CLP-A, CLP-B, CLP-F	CLPA and CLP-B may modulate regulatory genes such as FasL that regulate cell death. CLP-F downregulates the expression of the RANK protein.	[21,31,48]
Prevent tumor formation	CLP-A, CLP-C	CLP-A and CLP-C stimulate the expression of tumor suppressor TP53 and prevent tumor formation.	[21]
Induce expression of tumor necrosis factor (TNF)	CLP-A, CLP-C, CLP-E	CLP-A, CLP-C, and CLP-E stimulate the expression of TNF, which further induces tumor necrosis.	[21]
Inhibit cell proliferation	CLP-A, CLP-B, CLP-C, CLP-E	CLP-A, CLP-C, and CLP-E overexpress TP53BP2 and regulate the proliferation of the cells. Additionally, CLP-A and CLP-B modulate the AKT/JNK signaling pathway and inhibit cell proliferation.	[21,41,54]
Inhibit DNA synthesis	CLPs mixture	CLPs mixture inhibits DNA synthesis. The exact mechanism is not explained.	[13]
Regulate migration of tumor cells	CLP-A, CLP-C, CLP-E	CLP-A, CLP-C, and CLP-E induce overexpression of TP53BP2, which further regulates the migration of the cells.	[21]
